# Sotagliflozin, a Dual SGLT1/2 Inhibitor, Improves Cardiac Outcomes in a Normoglycemic Mouse Model of Cardiac Pressure Overload

**DOI:** 10.3389/fphys.2021.738594

**Published:** 2021-09-21

**Authors:** Sophia L. Young, Lydia Ryan, Thomas P. Mullins, Melanie Flint, Sarah E. Steane, Sarah L. Walton, Helle Bielefeldt-Ohmann, David A. Carter, Melissa E. Reichelt, Linda A. Gallo

**Affiliations:** ^1^School of Biomedical Sciences, The University of Queensland, St Lucia, QLD, Australia; ^2^Mater Research Institute-University of Queensland, Translational Research Institute, Woolloongabba, QLD, Australia; ^3^Cardiovascular Disease Program, Department of Physiology, Monash Biomedicine Discovery Institute, Monash University, Clayton, VIC, Australia; ^4^School of Veterinary Science, The University of Queensland, Gatton, QLD, Australia; ^5^Institute for Molecular Biosciences, The University of Queensland, St Lucia, QLD, Australia

**Keywords:** energy expenditure, energy intake, proximal tubular cell damage, cardiac hypertrophy, cardiovasclar disease, heart failure, hyperglycemia, high fat diet

## Abstract

Selective SGLT2 inhibition reduces the risk of worsening heart failure and cardiovascular death in patients with existing heart failure, irrespective of diabetic status. We aimed to investigate the effects of dual SGLT1/2 inhibition, using sotagliflozin, on cardiac outcomes in normal diet (ND) and high fat diet (HFD) mice with cardiac pressure overload. Five-week-old male C57BL/6J mice were randomized to receive a HFD (60% of calories from fat) or remain on ND for 12 weeks. One week later, transverse aortic constriction (TAC) was employed to induce cardiac pressure-overload (50% increase in right:left carotid pressure versus sham surgery), resulting in left ventricular hypertrophic remodeling and cardiac fibrosis, albeit preserved ejection fraction. At 4 weeks post-TAC, mice were treated for 7 weeks by oral gavage once daily with sotagliflozin (10 mg/kg body weight) or vehicle (0.1% tween 80). In ND mice, treatment with sotagliflozin attenuated cardiac hypertrophy and histological markers of cardiac fibrosis induced by TAC. These benefits were associated with profound diuresis and glucosuria, without shifts toward whole-body fatty acid utilization, increased circulating ketones, nor increased cardiac ketolysis. In HFD mice, sotagliflozin reduced the mildly elevated glucose and insulin levels but did not attenuate cardiac injury induced by TAC. HFD mice had vacuolation of proximal tubular cells, associated with less profound sotagliflozin-induced diuresis and glucosuria, which suggests dampened drug action. We demonstrate the utility of dual SGLT1/2 inhibition in treating cardiac injury induced by pressure overload in normoglycemic mice. Its efficacy in high fat-fed mice with mild hyperglycemia and compromised renal morphology requires further study.

## Introduction

Sodium-dependent glucose transporter (SGLT)-2 inhibitors have emerged as promising anti-diabetic agents, exerting rapid cardiovascular benefits beyond what would be expected from adequate glycemic control (Kosiborod et al., 2017; Fitchett et al., 2019; Wiviott et al., 2019). Irrespective of diabetic status, baseline HbA_1c_, or degree of glucose lowering, selective SGLT2 inhibitors reduce the risk of worsening heart failure or cardiovascular death in patients with existing heart failure and reduced ejection fraction (McMurray et al., 2019; Packer et al., 2020; Petrie et al., 2020; Anker et al., 2021). This led to recent FDA approvals for dapagliflozin and empagliflozin in the treatment of heart failure and reduced ejection fraction in adults with and without type 2 diabetes mellitus (DM).

SGLT2, located in the early kidney proximal tubule, is responsible for up to 97% of renal glucose reabsorption, while SGLT1, in the late proximal tubule, removes any remaining luminal glucose (Gallo et al., 2015). In patients with impaired renal function, selective SGLT2 inhibitors have reduced efficacy and, in all type 2 DM patients, modest effects on blood glucose lowering are observed: complete SGLT2 inhibition induces maximal glucosuria of only 40–60% due to compensatory upregulation of SGLT1-mediated kidney glucose reabsorption (Rieg et al., 2014; Gallo et al., 2015). SGLT1 is also the main transporter for intestinal glucose absorption (Gallo et al., 2015). Consequently, co-inhibition of SGLT1 and SGLT2 is being explored to provide greater blood glucose lowering effects and, potentially, cardiovascular benefits.

Sotagliflozin is a dual SGLT1/2 inhibitor, with approximately 20-fold selectivity for SGLT2 over SGLT1 (Gallo et al., 2015). While SGLT1 inhibition may pose a risk for intestinal glucose-galactose malabsorption, clinical trials in type 2 DM patients have demonstrated a favorable safety profile with sotagliflozin, as well as reductions in HbA_1c_, body weight, and blood pressure (Zambrowicz et al., 2012; Rosenstock et al., 2015). A recent study in patients with type 2 diabetes and worsening heart failure reported a 33% reduction in deaths from cardiovascular causes and hospitalizations and urgent visits for heart failure (Bhatt et al., 2021). It is unknown whether these benefits will extend to non-diabetic patients and/or those with preserved ejection fraction. Furthermore, while it is possible that dual SGLT1/2 inhibitors will offer vascular benefits analogous, or perhaps even superior, to selective SGLT2 inhibitors, SGLT1 is also expressed in the heart; most likely localized to heart capillaries (Banerjee et al., 2009; Kashiwagi et al., 2015; Vrhovac et al., 2015).

Increased myocardial SGLT1 expression has been reported in various disease states in humans (end-stage cardiomyopathy and type 2 DM) and mice (myocardial ischemia, type 2 DM, and glycogen storage cardiomyopathy) (Banerjee et al., 2009, 2010), but its functional role at this site remains largely unclear. It is therefore putative that the cardiac safety of therapeutic SGLT1/2 inhibition is examined upon states of cardiac compromise. Accordingly, we performed a descriptive assessment of cardiac outcomes in a mouse model of cardiac pressure overload treated with sotagliflozin. We further characterized kidney and metabolic outcomes in these mice.

## Materials and Methods

### Experimental Protocol

**FIGURE 1:**
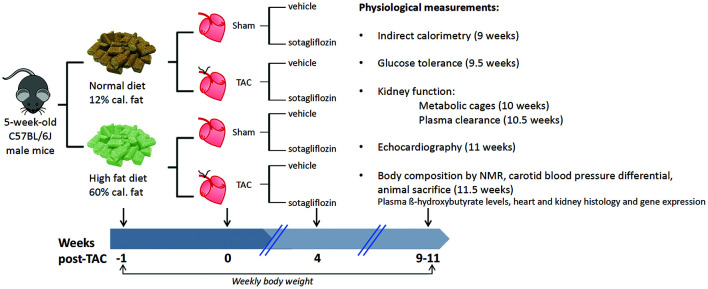
Group randomization and experimental flow chart. Five-week-old male C57BL/6J male mice were randomized to receive a HFD (60% of calories from fat) or remain on ND for 12 weeks. One week later, transverse aortic constriction (TAC) was employed to induce cardiac pressure-overload (50% increase in right:left carotid pressure versus sham surgery), resulting in left ventricular hypertrophic remodeling and cardiac fibrosis, albeit preserved ejection fraction. At 4 weeks into the dietary protocol, mice were treated for 7 weeks by oral gavage once daily with sotagliflozin (10 mg/kg body weight) or vehicle (0.1% tween 80). Body weight was measured weekly throughout the study period, and a range of physiological measurements were conducted in the last 2 weeks of study before animal sacrifice.

All procedures were performed with approval from The University of Queensland Animal Ethics Committee (#334/16) and in accordance with guidelines from the National Health and Medical Research Council of Australia. The study is reported in accordance with the ARRIVE guidelines. Four-week-old male C57BL/6J mice were purchased (Australian Resource Centre, Murdoch, Australia) and housed in an environmentally controlled room (constant temperature 22°C), with a 12 h light-dark cycle (lights on at 6:00 h and off at 18:00 h) and access to standard chow and RO water *ad libitum*. At 5 weeks of age, mice were randomized to receive a high fat diet (HFD) to exert a challenge on glucose metabolism and kidney function, or remain on normal diet (ND) for 12 weeks (Figure 1). The HFD was 21.7 MJ/kg; 60% of calories from fat, 20% from protein, 20% from carbohydrate, and 0.15% sodium (13-092, Specialty Feeds, Glen Forrest, Australia) and the ND was 14 MJ/kg; 12% of calories from fat, 23% from protein, 65% from carbohydrate, and 0.18% sodium. At 1 week into the dietary protocol, transverse aortic constriction (TAC) was employed to induce cardiac pressure-overload (Tan et al., 2018). Here, mice were anesthetized with a mixture of ketamine and xylazine (80/8 mg/kg) and a blunt 26-gage needle was positioned over the transverse aorta. The suture was tightened on the shaft of the needle to standardize the degree of aortic restriction and the needle immediately removed. Sham-operated mice were also included whereby the same procedures were carried out except for aortic constriction. At 4 weeks post-surgery, mice were treated for 7 weeks by oral gavage once daily with either sotagliflozin (SOTA, 10 mg/kg body weight) or vehicle (0.1% tween 80), resulting in eight groups (*n* = 6–7/group). Body weight and fasting blood glucose levels were monitored weekly throughout the study as per ethical requirements. At study completion (11.5 weeks post-TAC), body fat content was measured in conscious mice using nuclear magnetic resonance (Bruker’s Minispec MQ10, Houston, United States) before a tail blood sample was collected for the measurement of β-hydroxybutyrate levels (colorimetric assay kit, Cayman Chemical). Mice were then anesthetized with a mixture of ketamine and xylazine (80/8 mg/kg). The carotid systolic blood pressure differential was assessed using pressure catheters inserted into left and right carotid arteries and simultaneously recorded for 5 min (SPR1000; Millar Inc., Houston, United States). Mice were sacrificed at this time by cardiac puncture (11.5 weeks post-TAC). The heart, kidneys, and liver were excised and weighed. Hearts and kidneys were snap-frozen in liquid nitrogen or fixed in 10% neutral-buffered formalin for molecular and histological analyses, respectively. All physiological measurements described below were carried out in the last 2 weeks of study.

### Indirect Calorimetry

At 9 weeks post-TAC, an automated open-circuit indirect calorimetry system (TSE Systems, Bad Homburg, Germany) was used for the measurement of food and drink patterns, and energy balance, as previously described (Tan et al., 2018). This system comprised one environmental chamber, which allowed for individual monitoring of 12 mice in parallel with high resolution and precision. Mice were acclimatized to the new housing conditions for 7 days, followed by continuous recording for three consecutive days. Data were recorded for each animal every hour. To calculate day- and night-time averages, a single value per animal was used (the average of three 12-h cycles).

### Glucose Tolerance

At 9.5 weeks post-TAC, an oral glucose tolerance test (OGTT) was performed following a 5–6 h fast between 08:00 and 14:00 h using 50% w/v D-glucose solution (2 g/kg body weight) and a SensoCard glucometer (Tan et al., 2018).

### Kidney Function

At 10 weeks post-TAC, mice were weighed and placed individually into metabolic cages for 24 h urine collection (Tan et al., 2018). Mice were acclimatized to the metabolic cages by housing for a short period of daylight prior to the 24 h collection. An ELISA kit was used for the measurement of urinary albumin (Bethyl Laboratories, Montgomery, United States). At 10.5 weeks, glomerular filtration rate (GFR) was measured by plasma clearance of intravenously injected FITC-sinistrin (10 mg/100 g body weight dissolved in 0.9% NaCl). Briefly, FITC-sinistrin was retro-orbitally injected under brief isoflurane anesthesia (4%; 0.5–1.0 L/min, O_2_) and blood samples were collected from the tail vein in conscious mice at 3-, 5-, 7-, 10-, 15-, 35-, 56-, and 75-min post-injection. Plasma was fluorometrically measured (excitation 470 nm and emission 530 nm) and quantification achieved using a FITC-sinistrin standard curve of known concentrations. GFR was calculated using a two-compartment model (Rieg, 2013; Mullins et al., 2020).

### Echocardiography

At 11 weeks post-TAC, transthoracic echocardiography (M-mode) was performed in a blinded fashion by an experienced veterinarian (M.F.) using a Philips iE33 ultrasound machine with a 15 MHz linear transducer (Philips, Amsterdam, Netherlands) under 1.8% inhaled isoflurane anesthesia, as previously described (Tan et al., 2018). All data are the average of at least three consecutive beats.

### Heart and Kidney Histology

Paraffin sections (4 μm) were used for the blinded assessment of heart (cross-sectional plane) and kidney injury. All sections were visualized using an Aperio Slide Scanner. Myocyte size (average of 50 cross-sectional myocytes per animal) was quantified in the short axis plane in the left ventricle following Picrosirius red staining. A descriptive histopathology assessment was also performed by an expert pathologist (H.B.O.) using Masson’s Trichrome and Picrosirius red staining for hearts and Masson’s Trichrome, PAS, and H&E staining for kidneys. A score of 0 = within normal limits (wnl), 1 = minimal change, 2 = mild change, 3 = moderate change, and 4 = severe to very severe change.

### Heart and Kidney Real-Time qPCR

Total RNA was extracted from heart ventricles and kidney cortex using Trizol. cDNA was synthesized using iScript cDNA synthesis and real-time qPCR performed using pre-designed Taqman Gene Expression Assays for *Myh7* (Mm00600555_m1), *Nppa* (Mm01255747_g1), *Nppb* (Mm01255770_g1), *Acta1* (Mm00808218_g1), *Fibronectin* (Mm01256744_m1), *ColIV*α*1* (Mm01210125_m1), *Slc5a1* (Mm00451203_m1), *Slc5a2* (Mm00453831_m1), *Mct2* (Mm00441442_m1), and *Bdh1* (Mm00558330_m1) (Life Technologies, Mulgrave, VIC, Australia). Relative gene expression was quantified using the comparative threshold cycle (ΔΔCt.) with 18S rRNA (Life Technologies) as the endogenous multiplexed control. Ct. > 35 was considered not detectable (ND) and this applied to *Slc5a1* for some heart samples.

### Statistical Analyses

Data were analyzed by three-way ANOVA (SPSS, HFD × TAC × SOTA) or two-way ANOVA (GraphPad Prism 7, TAC x SOTA within each diet group). Upon significant interaction (or trend interaction where *P* = 0.05–0.07), data were split where appropriate and analyzed by two-way ANOVA or Tukey’s multiple comparisons test. Blood glucose levels during the OGTT and weekly body weight were analyzed by repeated measures two-way ANOVA and Tukey’s multiple comparisons test upon statistical interaction. All data were tested for normality using the Shapiro-Wilk test and log transformed for statistical analyses, where required. Data are expressed as means ± SD for normally distributed data or median ± IQR for not normally distributed data. *P* < 0.05 was considered significantly different.

## Results

### High Fat Diet Induced Mild Hyperglycemia, and Transverse Aortic Constriction Induced Cardiac Pressure Overload With Left Ventricular Hypertrophy and Pathology

**FIGURE 2:**
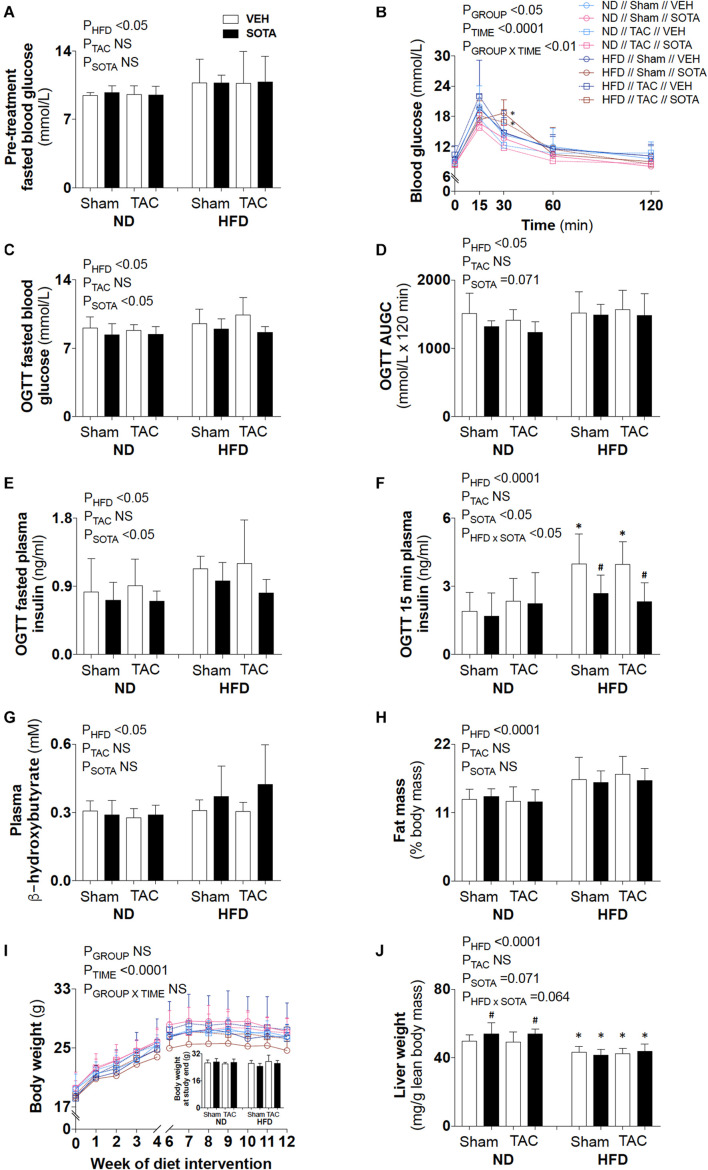
Circulating glucose, insulin, and ketone levels, and body composition in all groups. **(A)** Baseline (pre-treatment) fasted blood glucose levels, **(B–F)** post-treatment blood glucose profile, fasted blood glucose levels, area under the glucose curve (AUGC), fasted plasma insulin levels, and 15-min plasma insulin levels from the oral glucose tolerance test (OGTT), **(G)** plasma β-hydroxybutyrate levels, **(H)** fat mass relative to body mass, **(I)** weekly body weight and body weight at study end, and **(J)** liver weight. Data are means ± SD (*n* = 5–7/group). Interaction *P*-values by three-way ANOVA shown only when significant. *P < 0.05 vs. ND counterpart, ^#^*P* < 0.05 vs. VEH counterpart following significant interaction. NS, not significant.

HFD resulted in fasting hyperglycemia (Figures 2A,C), elevated area under the glucose curve during the OGTT (Figure 2D) and fasting and glucose-stimulated hyperinsulinemia (Figures 2E,F). Repeated measures two-way ANOVA during the OGTT revealed elevated blood glucose levels at 30 min in both SHAM and TAC sotagliflozin-treated HFD animals compared with ND counterparts (Figure 2B). At study end, circulating β-hydroxybutyrate levels (Figure 2G) and fat mass (Figure 2H) were increased in HFD versus ND mice, albeit unchanged total body weight between groups (Figure 2I). HFD resulted in a significant reduction in liver weight (Figure 2J). The effects of HFD on the heart are shown in Supplementary Figure 1 and Supplementary Table 1. Cardiac gene expression of *Nppa, Fibronection*, and *Bdh1* was increased in all HFD versus ND mice. Cardiomyocyte area was decreased by HFD in vehicle-treated animals but increased by HFD in sotagliflozin-treated animals. *Myh7* and *Nppb* gene expression were increased in HFD versus ND sotagliflozin-treated mice. There was minimal effect of HFD on echocardiography parameters; only LVPWd thickness was increased in sotagliflozin-treated TAC animals versus ND counterparts.

**FIGURE 3:**
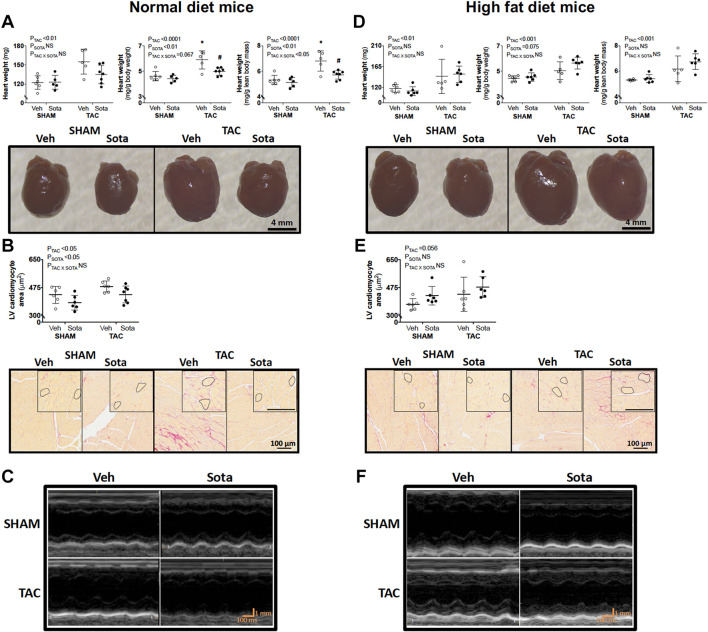
Heart size and cardiomyocyte area in mice with cardiac pressure overload treated with sotagliflozin, by ND and HFD. **(A,D)** Heart weight (absolute, relative to body weight, and relative to lean body mass), **(B,E)** cardiomyocyte area with higher magnification insets, and **(C,F)** echocardiography representatives in ND (left panel) and HFD (right panel) mice. Data are individual mice with means ± SD (*n* = 6–7/group). ^∗^*P* < 0.05 vs. SHAM counterpart, ^#^*P* < 0.05 vs. VEH counterpart by Tukey’s *post hoc* following significant interaction by two-way ANOVA. NS, not significant.

**FIGURE 4:**
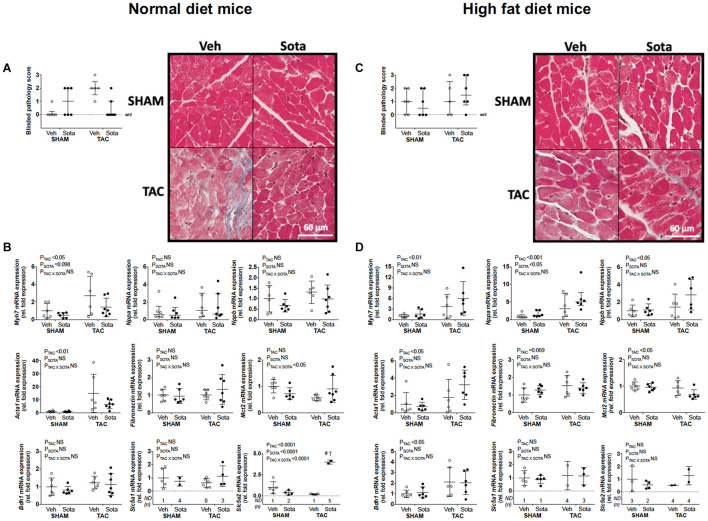
Cardiac histopathology and molecular markers of growth, fibrosis, and ketolysis in mice with cardiac pressure overload treated with sotagliflozin, by ND and HFD. **(A,C)** Pathology score (using trichrome- and picrosirius red-stained sections) assessed blindly by expert pathologist (H.B.O) and representatives of trichrome-stained sections, and **(B,D)** cardiac gene expression of *Myh7*, *Nppa*, *Nppb, Acta1, Fibronectin, Mct2, Bdh1, Slc5a1*, and *Slc5a2* in ND (left panel) and HFD (right panel) mice. Data are individual mice with means ± SD for *Myh7, Nppb, Acta1, Fibronectin, Mct2, Bdh1, Slc5a2*, and *Slc5a2* or median ± IQR for blinded pathology score and *Nppa* (*n* = 5–7/group). **(A,C)** (Pathology scores): Statistics were not performed on this categorical data. **(B)** (*Mct2* gene expression in ND mice): No differences observed with Tukey’s *post hoc* despite significant interaction by two-way ANOVA. **(B,D)** (*Nppa* gene expression): Statistics performed on log transformed data. ^#^*P* < 0.05 vs. VEH counterpart, ^†^*P* < 0.05 vs. SHAM counterpart following significant interaction. ND = not detected for *Slc5a1* and *Slc5a2* mRNA expression (i.e., Ct. > 35), NS, not significant; WNL, within normal limits for pathology score.

**FIGURE 5:**
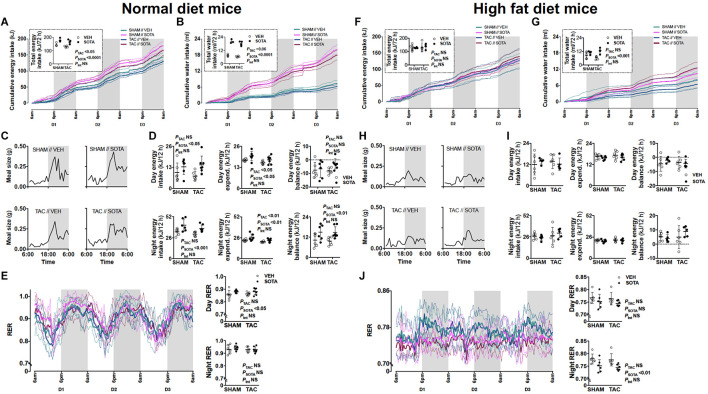
Food and water patterns, and energy balance in mice with cardiac pressure overload treated with sotagliflozin, by ND and HFD. **(A,F)** Energy intake, **(B,G)** water intake, **(C,H)** eating patterns (mean meal size each hour), **(D,I)** energy balance (excluding calorie loss with urinary glucose excretion, sotagliflozin: < 0.006 kJ/24 h, vehicle: < 0.00001 kJ/24 h), and **(E,J)** respiratory exchange ratio (RER) in ND (left panel) and HFD (right panel) mice. Data are means ± SD denoted by dotted lines or error bars (*n* = 5–7/group). NS, not significant. **(I)** No significant differences by two-way ANOVA.

TAC surgery successfully induced cardiac pressure overload in both ND and HFD mice (∼50% increase in right-to-left carotid systolic blood pressure), associated with left ventricular hypertrophic remodeling, albeit preserved fractional shortening and ejection fraction (Figure 3, Tables 1, 2 and Supplementary Figure 1, and Supplementary Table 1). Histological assessment of both trichrome- and picrosirius red-stained heart sections revealed a greater proportion of TAC mice with a degree of pathology including fibrosis, left ventricular myocyte hypertrophy, and/or scattered leukocyte infiltration compared with SHAM, albeit minimal to no evidence of inflammation at the time of mouse termination (Figures 4A,C). These TAC-induced effects on cardiac growth and pathology were most apparent in ND mice. At the molecular level, TAC surgery in HFD mice resulted in increased expression of all cardiac injury markers examined, including *Myh7, Nppa, Nppb, Acta1*, and *Fibronectin* but, in ND mice, only *Myh7* and *Acta1* were increased by TAC (Figures 4B,D). In ND vehicle-treated mice, TAC surgery decreased cardiac expression levels of the ketone body transporter, *Mct2*, albeit this was not significant upon *post hoc* testing (Figure 4B). In HFD vehicle- and sotagliflozin-treated mice, TAC surgery decreased cardiac *Mct2* and increased ketolytic enzyme, *Bdh1* (Figure 4D). The TAC-induced decrease in *Mct2* gene expression was most pronounced in sotagliflozin-treated animals (Supplementary Figure 1). Whole heart gene expression of *Slc5a1* and *Slc5a2* was low with some mice having undetectable levels (Figures 4B,D). There was no difference in *Slc5a1* between groups and, in ND mice that had detectable amounts of *Slc5a2*, expression levels were increased in sotagliflozin-treated TAC mice compared with both vehicle and SHAM counterparts (Figure 4B). In ND, but not HFD, mice TAC surgery reduced total energy and water intake, associated with reduced energy expenditure (Figure 5).

**TABLE 1:** Transcarotid pressure gradient and echocardiography parameters in ND mice.

	**SHAM**		**TAC**		***P-*value**	
	**VEH**	**SOTA**	**VEH**	**SOTA**	***TAC***	***SOTA***
Right:left carotid pressure	0.959 ± 0.046	0.790 ± 0.126	1.421 ± 0.175	1.335 ± 0.296	<0.001	NS
Heart rate (bpm)	494.69 ± 45.26	490.83 ± 62.32	473.26 ± 86.76	469.35 ± 61.65	NS	NS
LVIDd (mm)	3.626 ± 0.280	3.552 ± 0.392	3.351 ± 0.500	3.781 ± 0.328	NS	NS
LVAWd (mm)	0.844 ± 0.089	0.883 ± 0.166	1.102 ± 0.200	0.899 ± 0.146	<0.05	NS
LVPWd (mm)	0.987 ± 0.230	0.899 ± 0.105	1.546 ± 0.528	1.059 ± 0.154	<0.01	<0.05
LVIDs (mm)	2.574 ± 0.463	2.229 ± 0.467	2.195 ± 0.435	2.715 ± 0.454	NS	NS
LVAWs (mm)	1.013 ± 0.139	1.199 ± 0.145	1.365 ± 0.243*	1.172 ± 0.088	<0.05	NS
LVPWs (mm)	1.284 ± 0.319	1.381 ± 0.233	1.960 ± 0.551*	1.373 ± 0.277**^#^**	<0.05	NS
FS (%)	29.41 ± 9.18	37.79 ± 7.94	34.83 ± 6.71	28.54 ± 7.73	NS	NS
EF (%)	63.26 ± 12.07	74.88 ± 8.58	71.43 ± 8.04	62.38 ± 11.03	NS	NS

Data are means ± SD (n = 6–7/group). *P < 0.05 vs. SHAM counterpart, ^#^P < 0.05 vs. VEH counterpart by Tukey’s post hoc following significant interaction by two-way ANOVA. NS, not significant; LVIDd/s, left ventricular internal diameter in diastole/systole; LVAWd/s, left ventricular anterior wall thickness in diastole/systole; LVPWd/s, left ventricular posterior wall thickness in diastole/systole; FS, fractional shortening; EF, ejection fraction.

**TABLE 2:** Transcarotid pressure gradient and echocardiography parameters in HFD mice.

	**SHAM**		**TAC**		***P-*value**	
	**VEH**	**SOTA**	**VEH**	**SOTA**	***TAC***	***SOTA***
Right:left carotid pressure	0.988 ± 0.051	1.002 ± 0.017	1.460 ± 0.517	1.505 ± 0.476	<0.05	NS
Heart rate (bpm)	451.81 ± 80.31	447.84 ± 25.47	499.00 ± 96.81	493.45 ± 42.13	NS	NS
LVIDd (mm)	3.574 ± 0.386	3.498 ± 0.334	3.380 ± 0.156	3.653 ± 0.203	NS	NS
LVAWd (mm)	0.926 ± 0.104	0.868 ± 0.091	0.949 ± 0.079	1.061 ± 0.128	<0.05	NS
LVPWd (mm)	1.043 ± 0.230	1.097 ± 0.251	1.150 ± 0.205	1.201 ± 0.215	NS	NS
LVIDs (mm)	2.410 ± 0.542	2.537 ± 0.337	2.311 ± 0.202	2.656 ± 0.238	NS	NS
LVAWs (mm)	1.136 ± 0.158	1.065 ± 0.074	1.188 ± 0.114	1.235 ± 0.131	=0.054	NS
LVPWs (mm)	1.386 ± 0.339	1.413 ± 0.200	1.431 ± 0.236	1.535 ± 0.202	NS	NS
FS (%)	32.83 ± 11.17	27.41 ± 6.96	31.69 ± 3.02	27.27 ± 5.54	NS	NS
EF (%)	67.50 ± 15.07	60.71 ± 11.21	67.91 ± 4.35	60.8 ± 8.51	NS	NS

*Data are means ± SD (n = 5–6/group). No significant interactions observed by two-way ANOVA. NS, not significant; LVIDd/s, left ventricular internal diameter in diastole/systole; LVAWd/s, left ventricular anterior wall thickness in diastole/systole; LVPWd/s, left ventricular posterior wall thickness in diastole/systole; FS, fractional shortening; EF, ejection fraction.*

### Sotagliflozin Treatment Lowered Blood Glucose and Plasma Insulin Levels in Both Normal Diet and High Fat Diet Mice

Sotagliflozin reduced blood glucose and plasma insulin levels in both diet groups (Figures 2C–F). There was no effect of sotagliflozin on plasma β-hydroxybutyrate levels, body fat mass, or body weight (Figures 2G–I). Sotagliflozin resulted in a significant increase in liver weight in ND, but not HFD, mice (Figure 2J).

### Sotagliflozin Treatment Attenuated Cardiac Injury in Normal Diet, but Not High Fat Diet, Mice With Cardiac Pressure Overload

In ND mice, treatment with sotagliflozin reduced TAC-induced cardiac hypertrophy, including reductions in whole heart weight, cardiomyocyte size, and left ventricular anterior and posterior wall thickness (Figures 3A–C, Table 1, Supplementary Figure 1, and Supplementary Table 1). Blinded histological assessment by expert veterinary pathologist (H.B.O) revealed that TAC-induced pathology was mostly absent in ND mice treated with sotagliflozin (Figure 4A). These structural benefits were associated with an attenuation of the TAC-induced increase in cardiac *Myh7*, but not *Acta1*, gene expression (Figure 4B and Supplementary Figure 1). Treatment with sotagliflozin also had no effect on *Nppa, Nppb, Fibronectin*, *Mct2, Bdh1*, or *Slc5a1* expression levels (Figure 4B). For cardiac *Slc5a2* expression, five of seven ND TAC sotagliflozin-treated mice had undetectable levels but, of those that did, expression levels were significantly higher in these mice compared with both sham and vehicle counterparts (Figure 4B and Supplementary Figure 1). On the other hand, in HFD mice, sotagliflozin did not improve TAC-induced cardiac hypertrophy nor increased cardiac gene expression of all injury markers (Figures 3D–F, 4D, and Table 2). In fact, treatment with sotagliflozin in HFD TAC- and sham-operated mice increased relative gene expression of *Nppa* compared with vehicle (Figure 4D). In HFD mice, sotagliflozin did not attenuate the TAC-induced increase in *Bdh1* (Figure 4D and Supplementary Figure 1).

### Sotagliflozin Promoted Increased Energy Intake and Positive Energy Balance in Normal Diet, but Not High Fat Diet, Mice

In ND mice, treatment with sotagliflozin increased energy and water intake, with more “grazing” night-time eating patterns compared with vehicle (Figures 5A–C). This was associated with a smaller increase in energy expenditure (i.e., diet-induced thermogenesis), resulting in positive energy balance during night hours (Figure 5D). Adjustments for calorie loss via glucosuria (sotagliflozin mice: < 0.006 kJ/24 h, vehicle mice: < 0.00001 kJ/24 h) did not appreciably lower this positive energy balance in ND mice (data not shown). Increased day energy intake (Figure 5D) was associated with increased respiratory exchange ratio (RER), reflecting increased carbohydrate utilization, compared with vehicle-treated mice (Figure 5E). In HFD mice, sotagliflozin had no effect on energy intake or eating patterns but resulted in increased total water intake (Figures 5F–H). There was no effect of sotagliflozin on energy expenditure nor energy balance in HFD mice, although night-time RER was reduced, reflecting increased utilization of fat (Figures 5I,J). These changes to eating, drinking and/or overall metabolism were seen equally in SHAM and TAC animals.

### Lack of Cardio-Protection With Sotagliflozin in High Fat Diet Mice Was Associated With Proximal Tubule Injury

**FIGURE 6:**
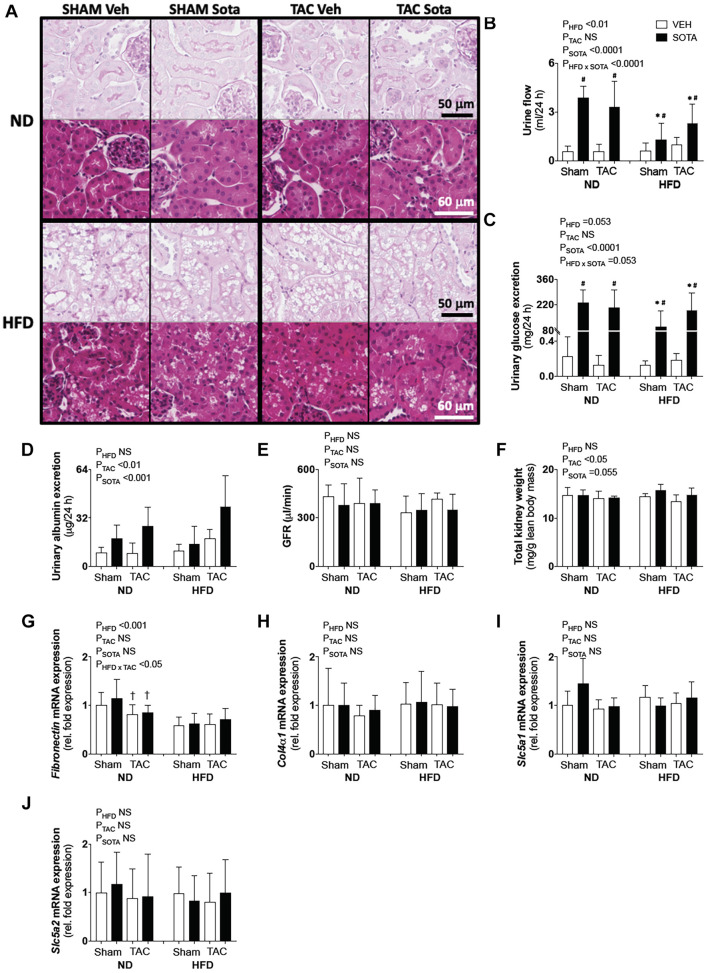
Kidney pathology in all groups. **(A)** Kidney histopathology representatives of periodic acid Schiff (PAS) and H&E staining used in descriptive histological assessments by expert pathologist (H.B.O), **(B)** 24-h urine production, **(C)** 24-h urinary glucose excretion, **(D)** albuminuria, **(E)** glomerular filtration rate (GFR), **(F)** kidney weight, **(G–J)** kidney gene expression of *Fibronectin*, *ColIVa1*, *Scl5a1*, and *Scl5a2*. Data are means ± SD (*n* = 5–7/group for all data, except for GFR where *n* = 3–5/group). Interaction *P*-values by three-way ANOVA shown only when significant or trend. ^∗^*P* < 0.05 vs. ND counterpart, ^†^*P* < 0.05 vs. SHAM counterpart, ^#^*P* < 0.05 vs. VEH counterpart following significant interaction. NS, not significant.

To help explain the differential cardiac effects of sotagliflozin between ND and HFD mice, we examined the status of the kidneys between these groups, as the kidneys are a primary target of sotagliflozin. Hence, a three-way ANOVA was conducted to allow for direct comparisons between ND and HFD mice. In all HFD mice, blinded histological assessment of PAS- and trichrome-stained sections, by expert veterinary pathologist (H.B.O.), revealed micro- and macrovacuolation of proximal tubular cells, largely restricted to segments 1 and 2 (S1/2) (Figure 6A). H&E staining did not reveal protein globules, implicating the vacuolation as being due to fat accumulation with mild cytoplasmic/membranous admixture in these HFD kidneys. The smooth, round appearance of the vacuoles is typical of cytoplasmic fat accumulation. The vast majority of glomeruli were considered normal. There was no kidney pathology induced or exacerbated by TAC or improved with sotagliflozin treatment. Associated with this kidney pathology was a lessened diuretic and glucosuric effect of sotagliflozin in HFD vs. ND mice (Figures 6B,C). HFD mice did not exhibit changes in albuminuria, GFR, kidney weight, or kidney gene expression of *ColIVa1*, *Slc5a1*, and *Slc5a2* but had reduced expression of *Fibronectin* (Figures 6D–J).

TAC surgery and sotagliflozin treatment both independently increased albuminuria; however, this effect of sotagliflozin was attributed to profound diuresis (Figure 6D). Kidney weight was reduced in all TAC mice, while sotagliflozin tended to increase in kidney weight (Figure 6F). In ND mice, there was a TAC-induced reduction in *Fibronectin* gene expression compared with SHAM (Figure 6G). Kidney mRNA expression of *Col4a1*, *Slc5a1*, and *Slc5a2* was not affected by TAC or sotagliflozin treatment (Figures 6H–J).

## Discussion

This study examined the cardiac effects of the novel glucose lowering agent, sotagliflozin, in a previously characterized murine model of cardiac pressure overload (Tan et al., 2018). In normoglycemic mice, treatment with sotagliflozin for 7 weeks attenuated cardiac hypertrophy and histological markers of cardiac fibrosis that were induced by mechanical pressure overload. This was associated with profound diuresis and glucosuria, and is the first study to demonstrate a cardio-protective effect with dual SGLT1/2 inhibition at therapeutic doses. These benefits were not seen in mildly hyperglycemic HFD animals with proximal tubular injury.

Dual inhibitors are under investigation for the treatment of diabetes and expected to yield greater glucose-lowering and pleiotropic benefits compared with selective SGLT2 inhibitors (Zambrowicz et al., 2012; Rosenstock et al., 2015). The cardio-protective effects of selective SGLT2 inhibitors are well established in patients with type 2 DM (Kosiborod et al., 2017; Fitchett et al., 2019; Wiviott et al., 2019) and in diabetic and non-diabetic patients with established heart failure and reduced ejection fraction (McMurray et al., 2019; Packer et al., 2020; Petrie et al., 2020; Anker et al., 2021). The cardio-protective effects of dual SGLT1/2 inhibition in patients with type 2 diabetes and worsening heart failure was recently reported (Bhatt et al., 2021), but it is unknown whether these benefits will extend to non-diabetic patients and/or those with preserved ejection fraction. In this study, TAC surgery induced a 50% increase in cardiac pressure overload in both ND and HFD mice, associated with left ventricular hypertrophic remodeling, fibrosis, leukocyte infiltration, albeit preserved ejection fraction. At the molecular level, TAC in HFD mice induced an upregulation of all cardiac injury markers examined, but many were surprisingly unaffected in ND mice. This may be attributed to differences in the resolution of specific molecular events between ND and HFD, noting that mice were euthanized and hearts collected for histology at 11.5 weeks post-TAC. Sotagliflozin treatment, commencing 4 weeks after the TAC procedure, overcame these mechanically induced structural risk factors for heart failure in ND mice. In a previous study using non-diabetic TAC mice with reduced ejection fraction, just 2 weeks’ treatment with empagliflozin, commencing 2–3 weeks post-TAC, attenuated a progressive decline in cardiac function (Byrne et al., 2017). There was no amelioration of cardiac fibrosis; however, this was not quantified, there was no molecular analyses, and the study lacked a sham-operated control group for reference. Indeed, a direct comparison to a selective SGLT2 inhibitor and tissue analyses at multiple time-points would have strengthened our study.

Elucidating the mechanisms for cardiac benefits with SGLT inhibition was not an objective of this study. However, this topic certainly warrants consideration. In our study, cardiac expression of the genes encoding SGLT1 and SGLT2 was very low and undetectable in some mice. Interestingly, a recent study reported that human and mouse hearts express a *Slc5a1* transcript variant resulting in an SGLT1-truncated protein that lacks transmembrane domains and residues (Ferte et al., 2021). Taken together, these data suggest that sotagliflozin is unlikely to mediate its myocardial improvements directly. There is, however, particular interest around changes to myocardial energetics, induced by systemic changes to substrate availability. In humans with and without type 2 DM, empagliflozin decreased RER indicating increased fatty acid utilization (Ferrannini et al., 2016). Reduced insulin levels with SGLT inhibition, as seen in this study, increases lipolysis and circulating free fatty acid levels. This, in turn, stimulates hepatic ketogenesis and increases circulating ketone levels as reported in both non-diabetic and diabetic subjects treated with SGLT2 inhibitors (Ferrannini et al., 2016; Polidori et al., 2018). With respect to the failing heart, evidence from both humans and animals shows that it becomes increasingly reliant on ketone bodies as a source of fuel, due to impaired free fatty acid and glucose oxidation (Aubert et al., 2016). Therefore, increased ketone availability under SGLT blockade is thought to “rescue” the failing heart, translating into cardiovascular benefits.

In a non-diabetic porcine model of heart failure, treatment with empagliflozin partially restored myocardial free fatty acid uptake and increased myocardial ketone body uptake by fourfold (Santos-Gallego et al., 2019). This was associated with increased myocardial activity and/or expression of ketone oxidation enzymes and ATP content as well as improved cardiac structure and contractile function. In non-diabetic rats with surgically induced myocardial infarction, empagliflozin treatment commencing either prior to, or after, surgery, similarly increased circulating ketone levels and myocardial ketone utilization (increased enzyme expression), associated with ameliorated cardiac hypertrophy, interstitial fibrosis, and oxidative stress and improved left ventricular function (Yurista et al., 2019). However, in Byrne et al. (2020) the beneficial effects of empagliflozin in non-diabetic TAC mice occurred in the absence of changes in blood ketone levels or cardiac ketone oxidation. Similarly, in normoglycemic TAC mice treated with sotagliflozin, we observed no changes in circulating ketone levels or cardiac expression of ketolysis-associated genes. Rather, a shift toward whole-body carbohydrate utilization and positive energy balance was seen with sotagliflozin in our study. This was likely mediated by increased energy intake and, hence, continuous carbohydrate fuel availability. Increased energy intake is seen in both humans and animals treated with SGLT inhibitors, as an adaptive response to glucosuria-induced energy loss (Ferrannini et al., 2015). In our HFD mice, however, there was no sotagliflozin-induced increase in energy intake and night-time RER decreased even further, beyond that caused by high fat feeding alone. Hence, a shift toward whole-body fatty acid utilization and elevated circulating ketones with SGLT inhibition may depend on restricted carbohydrate availability and may not be critical for, nor induce, myocardial benefits as seen in ND and HFD mice, respectively. In diet-induced obese rats treated with dapagliflozin, pair-feeding to match food intake of vehicle-treated animals resulted in a fourfold greater weight loss (Devenny et al., 2012). Therefore, additional cardiac benefits may become apparent with restricted food availability and a shift toward neutral or mild negative energy balance (irrespective of weight loss), which requires further study.

In the current study, cardiac benefits with sotagliflozin, seen in ND mice, were not evident in the HFD group. A key difference between the two groups was the reduced efficacy with respect to sotagliflozin-induced diuresis and glucosuria in HFD animals. While treatment with sotagliflozin lowered blood glucose levels in HFD mice, these were not normalized to ND levels and the resultant diuresis and glucosuria were only ∼50–65% of that in ND mice. This was associated with compromised kidney morphology in HFD mice, largely restricted to the renal site of drug action, i.e., the proximal tubule, which was not attenuated with sotagliflozin treatment. Fat deposition in proximal tubules is consistent with our previous observations in this model, where we also reported increased urinary excretion of the proximal tubule damage marker, KIM-1 (Tan et al., 2018). Others have also reported impaired sodium handling in high fat fed mice, suggestive of impaired proximal tubular function (Deji et al., 2009). Hence, the lack of cardiovascular benefit with sotagliflozin in our high fat fed TAC animals may arise from dampened proximal tubular drug action, i.e., ineffective diuresis and natriuresis, otherwise thought to improve cardiovascular hemodynamics, including reduced cardiac preload (Verma and McMurray, 2018). In the EMPA-REG trial, carried out in patients with type 2 DM and preserved renal function, 50% of the cardiovascular benefit was attributed to increased hematocrit, i.e., volume contraction (Inzucchi et al., 2018). Increased erythropoiesis has also been implicated; and this too can exert cardio-protective effects (Mazer et al., 2020). While our findings suggest that sotagliflozin was not effective for the treatment of cardiac injury in the high fat fed state, it is critical to note that in the DAPA-HF trial, both obese and type 2 DM patients equally benefitted with respect to cardiovascular outcomes (McMurray et al., 2019; Petrie et al., 2020). Our high fat fed mice, with mildly elevated blood glucose levels and compromised renal morphology, likely do not reflect the metabolic and kidney status of the trial patients. It is possible that greater cardioprotective benefits would have been observed with a more severe hyperglycemic model; however, improved cardiovascular outcomes with selective SGLT2 inhibition is seen across the spectrum of HbA1c in patients with and without diabetes (McMurray et al., 2019; Packer et al., 2020; Petrie et al., 2020; Anker et al., 2021), and a more severe model may have compromised renal morphology and drug action further.

As a dual inhibitor, sotagliflozin also mediates its blood glucose lowering effects at the level of small intestine, by delaying glucose absorption and reducing postprandial glucose (Zambrowicz et al., 2015). The intestinal mucosa of HFD animals, and thus drug action here, may have been compromised reflected by the higher blood glucose levels at 30 min into the OGTT in sotagliflozin-treated animals versus ND counterparts. However, given the glucose-independent cardiovascular benefits with selective SGLT2 inhibitors (McMurray et al., 2019; Petrie et al., 2020), it is unlikely that reductions in post-prandial glucose in ND mice mediated the cardiac benefits. Indeed, we cannot rule out other mechanisms from inhibited intestinal glucose absorption, such as increased glucagon-like peptide 1, which may have been compromised in HFD mice. It is also possible that the sotagliflozin treatment commenced too late in the HFD mice, after significant cardiac remodeling had already occurred. The temporal progression of cardiac injury was not examined in this study; and this warrants future investigation to ascertain optimal timing for treatment commencement.

Alternative mechanisms proposed for cardio-protection, for selective SGLT2 inhibitors at least, include other kidney effects that subsequently benefit heart function (e.g., changes in the renin angiotensin aldosterone system), inhibition of the sympathetic nervous system, and/or reduced cardiac cytosolic Na^+^ and restored Ca^2+^ handling through direct cardiac NHE-1 inhibition. In non-diabetic rodents, with either reduced or preserved ejection fraction, cardio-protection with empagliflozin was associated with reduced cardiac inflammation through attenuated activation of the nucleotide-binding domain-like receptor protein 3 (NLRP3) inflammasome, which was dependent on restoration of cardiac cytoplasmic Ca^2+^ levels (Byrne et al., 2020). While we observed improved cardiac histopathology, including attenuated leukocyte infiltration in the hearts of normoglycemic TAC mice treated with sotagliflozin, the suppression of specific inflammatory pathways was not investigated. Future studies should consider in-depth mechanistic exploration, including an examination of the relative contribution of tissue SGLTs to the observed effects.

This study demonstrated myocardial benefits with dual SGLT1/2 inhibition in normoglycemic mice with cardiac pressure overload. Cardiac benefits occurred in the absence of whole-body or cardiac-specific shifts toward fatty acid and/or ketone body utilization and, rather, were associated with profound diuresis and glucosuria. The lack of myocardial benefit in HFD mice suggests proximal tubular injury compromised drug-induced benefits.

## Data Availability Statement

The original contributions presented in the study are included in the article/Supplementary Material, further inquiries can be directed to the corresponding author/s.

## Ethics Statement

The animal study was reviewed and approved by The University of Queensland Animal Ethics Committee (#334/16).

## Author Contributions

LG and MR conceived the study. SY, LR, TM, MF, SS, SW, HB-O, DC, and LG conducted the experiments. SY, LR, DC, and LG analyzed the results. SY and LG wrote the manuscript. All authors reviewed the manuscript.

## Conflict of Interest

The authors declare that the research was conducted in the absence of any commercial or financial relationships that could be construed as a potential conflict of interest. The reviewer CQ declared a shared affiliation with one of the author SW to the handling editor at the time of the review.

## Publisher’s Note

All claims expressed in this article are solely those of the authors and do not necessarily represent those of their affiliated organizations, or those of the publisher, the editors and the reviewers. Any product that may be evaluated in this article, or claim that may be made by its manufacturer, is not guaranteed or endorsed by the publisher.

## Supplementary Material

Supplementary Figure 1 Cardiac parameters, total sodium intake, and weekly random blood glucose levels in all groups. **(a–c)** heart weight (absolute, relative to body weight, and relative to lean body mass), **(d)** cardiomyocyte area, **(e–m)** cardiac gene expression of *Myh7*, *Nppa*, *Nppb, Acta1, Fibronectin, Mct2, Bdh1, Slc5a1*, and *Slc5a2*, **(n)** total sodium intake, and **(o)** weekly random blood glucose levels. Data are means ± SD, except for **(f)** (*Nppa* gene expression) where data are median ± IQR and statistics performed on log transformed data (*n* = 4–7/group). Interaction *P*-values by three-way ANOVA shown only when significant or trend. ^∗^*P* < 0.05 vs. ND counterpart (^∗^*P* = 0.053–0.062 for cardiomyocyte area, ^∗^*P* = 0.098 for *Myh7*), ^†^*P* < 0.05 vs. SHAM counterpart, ^#^*P* < 0.05 vs. VEH counterpart (^#^*P* = 0.072 for *Mct2*) following significant interaction. ND = not detected for *Slc5a1* and *Slc5a2* mRNA expression (i.e., Ct. > 35), NS, not significant.
