# Supplementary figures and images for: Sotagliflozin, a Dual SGLT1/2 Inhibitor, Improves Cardiac Outcomes in a Normoglycemic Mouse Model of Cardiac Pressure Overload
Source: Front Physiol. 2021 Sep 21;12:738594. doi: 10.3389/fphys.2021.738594 (PMC8490778; doi:10.3389/fphys.2021.738594)

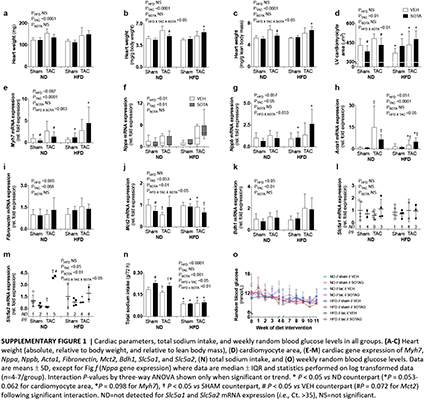

Supplement: Supplementary Figure 1 — Cardiac parameters, total sodium intake, and weekly random blood glucose levels in all groups. (a–c) heart weight (absolute, relative to body weight, and relative to lean body mass), (d) cardiomyocyte area, (e–m) cardiac gene expression of Myh7, Nppa, Nppb, Acta1, Fibronectin, Mct2, Bdh1, Slc5a1, and Slc5a2, (n) total sodium intake, and (o) weekly random blood glucose levels. Data are means ± SD, except for (f) (Nppa gene expression) where data are median ± IQR and statistics performed on log transformed data (n = 4–7/group). Interaction P-values by three-way ANOVA shown only when significant or trend. ∗P < 0.05 vs. ND counterpart (∗P = 0.053–0.062 for cardiomyocyte area, ∗P = 0.098 for Myh7), †P < 0.05 vs. SHAM counterpart, #P < 0.05 vs. VEH counterpart (#P = 0.072 for Mct2) following significant interaction. ND = not detected for Slc5a1 and Slc5a2 mRNA expression (i.e., Ct. > 35), NS, not significant. [file Image_1.tiff]
