# Supplementary material for: Sotagliflozin, a Dual SGLT1/2 Inhibitor, Improves Cardiac Outcomes in a Normoglycemic Mouse Model of Cardiac Pressure Overload
Source: Front Physiol. 2021 Sep 21;12:738594. doi: 10.3389/fphys.2021.738594 (PMC8490778; doi:10.3389/fphys.2021.738594)
Supplement: Supplementary file 2 [file Table_1.DOCX]

|  | ND | | | | HFD | | | |  | *P value* | | | |  | |
| --- | --- | --- | --- | --- | --- | --- | --- | --- | --- | --- | --- | --- | --- | --- | --- |
|  | SHAM VEH | SHAM SOTA | TAC VEH | TAC SOTA | SHAM VEH | SHAM SOTA | TAC VEH | TAC SOTA | *HFD* | | *TAC* | *SOTA* | *SIG. INT.* | |  |
| Right:left carotid pressure | 0.959 ± 0.046 | 0.790 ± 0.126 | 1.421 ± 0.175 | 1.335 ± 0.296 | 0.988 ± 0.051 | 1.002 ± 0.017 | 1.460 ± 0.517 | 1.505 ± 0.476 | NS | | <0.001 | NS | NS | |  |
| Heart rate (bpm) | 494.69 ± 45.26 | 490.83 ± 62.32 | 473.26 ± 86.76 | 469.35 ± 61.65 | 451.81 ± 80.31 | 447.84 ± 25.47 | 499.00 ± 96.81 | 493.45 ± 42.13 | NS | | NS | NS | NS | |  |
| LVIDd (mm) | 3.626 ± 0.280 | 3.552 ± 0.392 | 3.351 ± 0.500 | 3.781 ± 0.328^#^ | 3.574 ± 0.386 | 3.498 ± 0.334 | 3.380 ± 0.156 | 3.653 ± 0.203^#^ | NS | | NS | NS | TACxSOTA | |  |
| LVAWd (mm) | 0.844 ± 0.089 | 0.883 ± 0.166 | 1.102 ± 0.200^†^ | 0.899 ± 0.146 | 0.926 ± 0.104 | 0.868 ± 0.091 | 0.949 ± 0.079 | 1.061 ± 0.128^†^ | NS | | <0.01 | NS | HFDxTACxSOTA | |  |
| LVPWd (mm) | 0.987 ± 0.230 | 0.899 ± 0.105^#^ | 1.546 ± 0.528^†^ | 1.059 ± 0.154^†#^ | 1.043 ± 0.230 | 1.097 ± 0.251 | 1.150 ± 0.205^†^ | 1.201 ± 0.215^*^ | NS | | <0.01 | NS | HFDxSOTA | |  |
| LVIDs (mm) | 2.574 ± 0.463 | 2.229 ± 0.467 | 2.195 ± 0.435 | 2.715 ± 0.454^#^ | 2.410 ± 0.542 | 2.537 ± 0.337 | 2.311 ± 0.202 | 2.656 ± 0.238^#^ | NS | | NS | NS | TACxSOTA | |  |
| LVAWs (mm) | 1.013 ± 0.139 | 1.199 ± 0.145^#^ | 1.365 ± 0.243^†^ | 1.172 ± 0.088 | 1.136 ± 0.158 | 1.065 ± 0.074 | 1.188 ± 0.114 | 1.235 ± 0.131^†^ | NS | | <0.01 | NS | HFDxTACxSOTA | |  |
| LVPWs (mm) | 1.284 ± 0.319 | 1.381 ± 0.233 | 1.960 ± 0.551^†^ | 1.373 ± 0.277^#^ | 1.386 ± 0.339 | 1.413 ± 0.200 | 1.431 ± 0.236 | 1.535 ± 0.202 | NS | | <0.05 | NS | HFDxTACxSOTA | |  |
| FS (%) | 29.41 ± 9.18 | 37.79 ± 7.94 | 34.83 ± 6.71 | 28.54 ± 7.73 | 32.83 ± 11.17 | 27.41 ± 6.96 | 31.69 ± 3.02 | 27.27 ± 5.54 | NS | | NS | NS | NS | |  |
| EF (%) | 63.26 ± 12.07 | 74.88 ± 8.58 | 71.43 ± 8.04 | 62.38 ± 11.03 | 67.50 ± 15.07 | 60.71 ± 11.21 | 67.91 ± 4.35 | 60.8 ± 8.51 | NS | | NS | NS | NS | |  |

**Supplementary Table 1. Transcarotid pressure gradient and echocardiography parameters in all groups.**

Only significant (*P*<0.05) interactions by three-way ANOVA are shown in *Sig. Int.* column. * *P* < 0.05 *vs* ND counterpart, † *P* < 0.05 *vs* SHAM counterpart, # *P* < 0.05 *vs* VEH counterpart following significant interaction. NS=not significant, SIG. INT.=significant interaction.
